# Comparing technology acceptance of K‐12 teachers with and without prior experience of learning management systems: A Covid‐19 pandemic study

**DOI:** 10.1111/jcal.12552

**Published:** 2021-08-19

**Authors:** Muhterem Dindar, Anna Suorsa, Jan Hermes, Pasi Karppinen, Piia Näykki

**Affiliations:** ^1^ Learning and Educational Technology Research Unit Faculty of Education, University of Oulu Oulu Finland; ^2^ History, Culture and Communication Studies Faculty of Humanities, University of Oulu Oulu Finland; ^3^ Department of Marketing, Management and International Studies Oulu Business School, University of Oulu Oulu Finland; ^4^ Department of Organisational Behaviour and Marketing Faculty of Economic Sciences and Management, Nicolaus Copernicus University Toruń Poland; ^5^ Oulu Advanced Research on Service and Information Systems Faculty of Information Technology and Electrical Engineering, University of Oulu Oulu Finland; ^6^ Faculty of Education and Psychology University of Jyväskylä Jyväskylä Finland

**Keywords:** Covid‐19, K‐12 education, online education, technology acceptance, UTAUT

## Abstract

Covid‐19 pandemic has caused a massive transformation in K‐12 settings towards online education. It is important to explore the factors that facilitate online teaching technology adoption of teachers during the pandemic. The aim of this study was to compare Learning Management System (LMS) acceptance of Finnish K‐12 teachers who have been using a specific LMS as part of their regular teaching before the Covid‐19 pandemic (experienced group) and teachers who started using it for emergency remote teaching during the pandemic (inexperienced group). Based on the Unified Theory of Acceptance and Use of Technology framework, a self‐report questionnaire was administered to 196 teachers (*n*_experienced_ = 127; *n*_inexperienced_ = 69). Our findings showed no difference between the two groups of teachers in terms of performance expectancy, effort expectancy, LMS self‐efficacy and satisfaction. However, the experienced group had higher behavioural intention to use LMS in the future, reported receiving higher online teaching support and displayed higher online teaching self‐efficacy in terms of student engagement, classroom management, instructional strategies and ICT skills. For the experienced group, the most significant predictor of satisfaction with LMS was performance expectancy whereas for the inexperienced group, it was the effort expectancy. In terms of behavioural intention to use LMS in the future, the most significant predictor was the performance expectancy for both groups. Further, support was also a significant predictor of behavioural intention for the inexperienced group. Overall, our findings indicate that teachers should not be regarded as a unified profile when managing technology adoption in schools.

## 1 INTRODUCTION

Worldwide, there has been a growing emphasis on developing digital competencies in school settings (Siddiq et al., 2016). It is argued that education systems should develop digital skills of individuals to help them cope with the demands of an increasingly digital world (Voogt & McKenney, 2017). Thus, schools continually invest in Information and Communication Technologies (ICT), and teachers are encouraged to take advantage of technology in their teaching practices (Valtonen et al., 2017). However, using technology to facilitate meaningful learning has been challenging for many teachers, which can be seen in the limited use of technology in their classrooms (Fraillon et al., 2014; OECD, 2015). Therefore, developing knowledge and skills of teachers in utilizing technology effectively in their classrooms has been an important agenda for governments across the globe (Chen & Jang, 2014).

A significant number of studies has explored the promise and challenges of teachers' ICT usage in school settings through a variety of frameworks (Scherer et al., 2019). For example, the technological pedagogical content knowledge (TPACK) framework has been introduced to conceptualize how technology can be blended with pedagogical practices in the classrooms (Mishra & Koehler, 2006). In addition, several technology acceptance frameworks have been utilized to explain the factors that contribute to teachers' technology acceptance. Among those, Unified Theory of Acceptance and Use of Technology (UTAUT) has been a prominent framework in studying teachers' behavioural intention to use technology for teaching and learning (Chao, 2019). UTAUT originally comprises four key factors (performance expectancy, effort expectancy, social influence, facilitating conditions) and several mediators (gender, age, experience and voluntariness of use) that impact the behavioural intention to use a specific technology (Venkatesh et al., 2003). UTAUT was able account for 70% of the variance in intention to use, which has cemented the model as a gold standard for measuring ICT acceptance and usage varying from, for example, mobile learning (Chao, 2019) to telepresence robot in an educational setting (Han & Conti, 2020). UTAUT has been extensively applied in university settings specifically among pre‐service teachers and university lecturers (Baydas & Goktas, 2017; Garone et al., 2019). However, it has been applied to a limited extend in studying technology acceptance of in‐service teachers in K‐12 education (Wong, 2016).

The ongoing Covid‐19 pandemic has caused unforeseen interruptions in education worldwide. Many countries have responded to the pandemic crisis by switching to online distance education. In general, K‐12 schooling systems are designed for face‐to‐face education. Thus, the urgent switch from face‐to‐face to online distance education has created a state of chaos in many schools. School administrators, teachers and parents have been struggling to facilitate meaningful and effective learning experiences throughout the chaos (Richmond et al., 2020). In a short period of time, schools have had to adopt a variety of technologies [e.g., online teaching tools and Learning Management Systems (LMS) to continue their education]. During the transition to online remote education, teachers have faced multiple challenges such as lack of technological infrastructure and support, inexperience with digital technologies and lack of online teaching skills (Khlaif et al., 2020). This has caused immense amounts of workload and stress on teachers (Marek et al., 2020). Consequently, students have expressed several concerns about the quality of online teaching during the pandemic (Patricia Aguilera‐Hermida, 2020; Perrotta, 2020). Further, studies have found significant differences between the schools in terms of the quality of the pandemic‐time education (Maity et al., 2020). Considering these findings, it is important to investigate the factors that facilitate effective technology integration in schools during this extraordinary time. Drawing on this, the current study compared technology acceptance of two groups of Finnish K‐12 teachers. The first group has been using a specific LMS (i.e., Qridi) before the pandemic as a support to their classroom teaching, and continued using the LMS when the schools transformed to mostly online teaching due to pandemic. This group is named as experienced group in this study. The second group started using the Qridi LMS for emergency online teaching due to the pandemic. This group is named as inexperienced group in this study. Based on the UTAUT framework (Venkatesh et al., 2003), the study explores whether the two groups differ from each in terms of performance expectancy, effort expectancy, Qridi LMS self‐efficacy, support, satisfaction, online teaching self‐efficacy and behavioural intention to use LMS in the future. Further, the study investigates the factors that predict satisfaction and behavioural intention to use LMS for each group. The existing literature on technology acceptance mostly deals with teachers' use of technology in the classroom as a pedagogical support rather than being the actual teaching medium (Scherer & Teo, 2019). Thus, there is dearth of research on K‐12 in‐service teachers' technology use in solely online teaching condition. The current study addresses this gap through studying Finnish K‐12 teachers' technology acceptance for online teaching. Further, the current study contributes to the literature by exploring how previous experience with LMS affects technology acceptance for emergency online teaching.

## 2 LITERATURE REVIEW

### 2.1 Teachers' technology acceptance and UTAUT

A prominent framework in studying teachers' technology acceptance has been Technology Acceptance Model (TAM) (Davis et al., 1989). TAM asserts that behavioural intention to use a specific technology is influenced by two core beliefs: perceived usefulness and perceived ease of use. Perceived usefulness refers to the belief that using a specific technology will improve work performance (Davis, 1989). Perceived ease of use refers to the belief whether using a particular technology is free of effort (Davis et al., 1989). Venkatesh et al. (2003) extended the TAM with new constructs from other technology acceptance frameworks (i.e., Theory of Reasoned Action, Theory of Planned Behaviour, Motivational Model, Model of PC utilization, Socio‐cognitive theory, Diffusion of Innovations) and developed UTAUT. According to UTAUT, performance expectancy, effort expectancy, facilitating conditions and social influence are the main predictors of technology acceptance that is operationalized as behavioural intention to use technology. Performance expectancy encompasses perceived usefulness in TAM, and is the belief about the extend the technology will improve the job performance (Venkatesh et al., 2003). Effort expectancy is based on the *perceived ease of use* in TAM, and refers to the perceptions about how easy it is to use a technology (Venkatesh et al., 2012). Facilitating conditions refers to the beliefs regarding whether technical infrastructure, relevant training and support are provided to the individuals (Venkatesh et al., 2003). Social influence comprises beliefs about the degree which important others value or suggest the use of a specific technology (Venkatesh et al., 2012).

To date, UTAUT model has been tested in diverse educational contexts and extended with new factors. Specifically, self‐efficacy have emerged as significant variable that influence technology adoption (Venkatesh et al., 2012). In technology acceptance literature, self‐efficacy has been seen as a general or a targeted concept. As a general concept, self‐efficacy refers to teachers' self‐perceptions about their competency in employing effective pedagogical activities with the support of technology (Tondeur et al., 2020). According to this perspective, self‐efficacy does not imply a specific technology, but it is rather teachers' overall ICT competencies. For example, research on TPACK framework underlines the importance of general ICT competencies of teachers' in their educational practices (Mishra & Koehler, 2006). From a targeted perspective, self‐efficacy refers to the teachers' beliefs about their capability in using a specific educational technology (Kemp et al., 2019). Regardless of general or targeted, teachers' ICT self‐efficacy have been generally found to influence their technology acceptance and use (Barton & Dexter, 2020; Chiu, 2017; Hong et al., 2020; Long et al., 2018). However, studies on the interplay of self‐efficacy and technology acceptance in schools either solely focus on general self‐efficacy or targeted self‐efficacy. Therefore, a current gap in the literature is to explore how general and targeted self‐efficacy together impact technology acceptance in K‐12 settings.

Recent advancements in UTAUT research have also shown that affective factors such as pleasure, enjoyment and satisfaction influence technology acceptance (Ching‐Ter et al., 2017; Kemp et al., 2019; Venkatesh et al., 2012). For example, satisfaction has been found to predict future intention to use mobile learning (Chao, 2019). Further, satisfaction has been found to predict actual use of e‐learning environments along with behavioural intention (Mohammadi, 2015). Perceived enjoyment was a significant predictor of intention to use technology among pre‐service teachers in their prospective teaching (Teo & Noyes, 2011). Enjoyment and interest were further related with LMS and e‐learning environment usage among higher education students (Ching‐Ter et al., 2017; Khechine et al., 2020). Despite such findings, there is dearth of research on how affective factors, specifically satisfaction, influence technology acceptance among in‐service teachers.

Technology acceptance is not a one‐time process, and occurs over time (Blau & Shamir‐Inbal, 2017). Thus, it is important to study technology acceptance by focusing on users who are at different of their technology experience and acceptance. This is because actual experience with a technology might change individuals' beliefs and perceptions about the necessity the technology over time (Johnson et al., 2014). Nevertheless, only few studies investigated technology acceptance among different teacher groups in terms of their adoption stage. For example, Šumak and Šorgo (2016) have found significant differences between teachers who were either pre‐ or post‐adopters of interactive whiteboards. For pre‐adopters, the social influence had a stronger impact on behavioural intention, and performance expectancy had stronger impact on attitude to use whiteboards compared to the post‐ adopters. For post‐adopters, facilitating conditions had stronger impact on actual use of interactive whiteboards than pre‐adopters. In another study, Šumak et al. (2017) investigated teachers' technology acceptance among the prospective, existing and former users of interactive whiteboards. Their findings revealed that existing users displayed higher scores in performance expectancy, effort expectancy and management support. No difference was observed between former and prospective users. Ursavaş et al. (2019) compared technology acceptance of pre‐ and in‐service teachers. For pre‐service teachers, attitude towards using technology was the strongest predictor of behavioural intention followed by subjective norms, perceived ease of use and perceived usefulness. For the in‐service teachers, the strongest predictor of behavioural intention was attitude followed by perceived usefulness, perceived ease of use and subjective norms. In their longitudinal study, Pynoo et al. (2011) have explored secondary school teachers' acceptance of digital learning environments at three different time points. It has been found that performance expectancy was the primary predictor of behavioural intention at the beginning, but it was marginally significant at the end. Effort expectancy was not a significant predictor of behavioural intention at the beginning of the study, but it was the strongest predictor of behavioural intention at the end. No direct effect of facilitating conditions were found on technology acceptance. Overall, the limited number of findings show that teachers' perceptions at the different technology acceptance dimensions might shift in later stages of technology adoption depending on their experience with the system. Nevertheless, the current literature offers little understanding about teachers' technology acceptance in extraordinary circumstances such as Corona pandemic. Further, studies comparing technology acceptance of teachers who are at different stages of adoption process mostly measured technology acceptance in terms of behavioural intention (Scherer & Teo, 2019). However, the affective outcomes of technology acceptance (e.g., satisfaction) across different teacher profiles is yet to be explored.

The ongoing Covid‐19 pandemic has triggered a new phase of technology use in educational settings. On a very short notice, schools across the world had to adopt online tools and environments to continue their education. Regardless of whether they have the relevant skills or not, many K‐12 teachers have found themselves teaching online as an emergency response to the pandemic. Such abrupt switching to online education is worthy of investigation from multiple aspects in terms of technology acceptance. For example, it would be interesting to know to what extend technology acceptance in emergency teaching conditions differ from technology acceptance in regular teaching conditions (e.g., before the Covid‐19 pandemic). Further, comparing the factors that influence technology acceptance for both teaching conditions deserves attention. It is known for long that past experiences with a technology influences individuals' intention to use it (Fishbein & Ajcen, 1975). However, as summarized previously, only few studies have dealt with comparing technology acceptance of K‐12 teachers in terms of their previous experiences. There is dearth of research on the relationship between technology acceptance constructs among the teachers with differing technology experience. Considering this, the current study compares technology acceptance of Finnish K‐12 teachers who have been using Qridi LMS before the Covid‐19 pandemic (experienced group) and who started Qridi LMS due to Covid‐19 pandemic (inexperienced group).

## 3 RESEARCH QUESTIONS

*RQ1*. Are there differences between the experienced and inexperienced teacher groups in terms of their LMS acceptance: (a) performance expectancy, (b) effort expectancy, (c) support, (d) Qridi LMS self‐efficacy, (e) satisfaction, (f) behavioural intention and (g) online teaching self‐efficacy?

*RQ2*. What are the predictors of satisfaction and behavioural intention to use LMS for the experienced group and the inexperienced group of teachers?

## 4 METHODOLOGY

### 4.1 Procedure and participants

Finnish K‐12 schools transformed from face‐to‐face education to mostly online teaching on 17 March 2020. Online teaching period ended on 14 May 2020. During this period, face‐to‐face teaching continued with a small minority of students with special education needs and students between first and third grade, who could not get support for online learning at home. Data collection in the current study started on 10 May 2020 and ended on 5 June 2020. In collaboration with Qridi Company, Finnish K‐12 teachers who have been using the Qridi LMS were contacted through their e‐mail addresses. Volunteering teachers participated in the study by clicking on the online survey link provided in the e‐mail. Among the participants, five teachers were randomly chosen, and each was given a 20 EUR gift card from a bookstore. In total, 196 teachers completed the online survey in full. The mean age of the participants was 44 (*SD* = 9.6). A total of 25 of them were male, and 169 of them were female. A total of 130 participants were primary school teachers and 65 of them were subject teachers. Participants represented 19 provinces of Finland. Majority of them were from North‐Ostrobothnia (*n* = 85; % = 43) followed by Uusimaa (*n* = 36; % = 18), Pirkanmaa (*n* = 15; % = 8), North Savo (*n* = 12; % = 6), Southwest (*n* = 9; % = 5) and Lapland (*n* = 7; % = 4). A total of 127 of the participants have been using the Qridi platform before the Covid‐19 pandemic, and 69 of them have started using the platform after the Covid‐19 pandemic.

### 4.2 Measures and instruments

In UTAUT literature, main variables of interest have been behavioural intention, satisfaction, performance expectancy, effort expectancy, self‐efficacy, social influence and facilitating conditions (Garone et al., 2019; Venkatesh et al., 2012). In the current study, we employed questionnaires to measure behavioural intention, satisfaction, performance expectancy and effort expectancy. Further, we used two different self‐efficacy questionnaire that measure teachers both targeted (i.e., Qridi LMS) and general (i.e., online teaching) self‐efficacy. Participants of the current study had no other option than online teaching due to the pandemic. Social influence (i.e., beliefs of significant others on using a technology) is a redundant factor in such an obligatory online teaching condition. Therefore, social influence was left out in this study. Facilitating conditions factor in UTAUT underlines two issues in terms of technology acceptance: existing infrastructure and support (Venkatesh et al., 2003). All participants in this study were provided Qridi LMS. Therefore, we only measured the support aspect of facilitating conditions.

### 4.3 LMS technology acceptance

Teachers' LMS acceptance was measured with a questionnaire adapted from Chao (2019). The Likert type questionnaire measured teachers' technology acceptance in the following dimensions: Effort Expectancy (five items; e.g., ‘*I find Qridi easy to use*’), Performance Expectancy (four items; e.g., ‘*Using Qridi has enhanced my effectiveness in online teaching*’), Satisfaction (five items; e.g., ‘*I have been pleased with Qridi*’), Qridi LMS self‐efficacy (three items, e.g., ‘*I am confident of using Qridi even if there is no one around to show me how to do it*’) and Behavioural Intention (three items; e.g., ‘*I plan to use Qridi in the future*’). The questionnaire displayed good internal consistency across its dimensions according to Cronbach's Alpha scores (Effort expectancy = 0.88; Performance expectancy = 0.82; Satisfaction = 0.90; Qridi self‐efficacy = 0.81; Behavioural Intention = 0.88).

A three‐item questionnaire was developed to measure the extend of *support* provided to the teachers for their online teaching. The items of the questionnaire were ‘*I am provided support when solving problems in online teaching*’, ‘*Resources (e.g., tools, software, guidelines) about online teaching are made available to me*’, and ‘*I am provided opportunities for personal development in online teaching*’. Internal consistency of the questionnaire was 0.676. A Principal Component Analysis was conducted to examine the factor structure of the questionnaire. The single factor structure (Kaiser Meyer Olkin Measure of Sampling Adequacy = 0.621; Bartlett's test of sphericity = 102.1; *p* < 0.001)) explained 61% of the total variance. The component matrix scores of the items were between 0.677 and 0.816. The item communality scores ranged between 0.458 and 0.712.

In total there were 23 items in the LMS technology acceptance questionnaire sheltering all the constructs summarized above. Questionnaire items were answered on a five‐point scale. Answers varied between ‘Strongly disagree’ to ‘Strongly agree’. The whole questionnaire had a Cronbach's Alpha value of 0.92.

## 5 ONLINE TEACHING SELF‐EFFICACY QUESTIONNAIRE

The questionnaire was originally developed by Robinia (2008), and measures teachers' online teaching self‐efficacy under four dimensions with 32 items. These dimensions are Student engagement (e.g., ‘*How much can you do to motivate students who show low interest in online work?*’), Instructional strategies (e.g., ‘*How much can you do to use a variety of assessment strategies for an online course?*’), Classroom management (e.g., ‘*How much can you do to get students to follow the established rules for assignments and deadlines during an online class?*’) and ICT efficacy (e.g., ‘*How well can you navigate the technical infrastructure at your institution to successfully create an online course?*’). Each dimension sheltered eight items. In the current sample, the questionnaire dimensions displayed good internal consistency in terms of Cronbach's alpha scores (Student Engagement = 0.79; Instructional strategies = 0.82; Classroom management = 0.73; ICT efficacy = 0.804). The Cronbach's Alpha score for the whole questionnaire was 0.92.

### 5.1 Data analysis

Each variable of interest in the current study was measured with multiple items. An initial step prior to the statistical analyses was to calculate mean scores of items belonging to each variable. Based on the mean scores, normal distribution of the variables across the two teacher groups (i.e., experienced and inexperienced) were checked. Table 1 presents Skewness and Kurtosis results.

**TABLE 1:** Distribution of variables across the comparison groups

	Experienced group		Inexperienced group	
	Skewness	Kurtosis	Skewness	Kurtosis
Effort expectancy	−0.823	1.446	−1.001	1.057
Performance expectancy	−0.568	−0.475	−0.656	0.072
Qridi self‐efficacy	−0.521	0.139	−1.134	2.476
Satisfaction	−0.409	−0.334	−0.836	0.612
Behavioural intention	−1.603	1.929	−1.303	2.426
Support	−1.254	1.64	−0.643	0.197
Student engagement self‐efficacy	0.287	0.037	0.895	1.668
Classroom management self‐efficacy	0.09	−0.283	0.586	0.244
Instructional strategies self‐efficacy	0.012	−0.311	−0.29	1.282
ICT self‐efficacy	−0.325	−0.117	−0.534	0.843

Based on the results presented in Table 1, it can be claimed that all variables displayed normal distribution. In order to answer the first research question, is there differences between the experienced and inexperienced groups of teachers in terms of their LMS acceptance, independent samples *t* tests were conducted for each LMS acceptance dimension (i.e., Effort expectancy, Performance expectancy, Qridi self‐efficacy, Support, Satisfaction, Behavioural Intention and online teaching self‐efficacy (i.e., Student engagement, Classroom management, Instructional strategies and ICT self‐efficacy). The analysis proceeded to explore the predictors of satisfaction and behavioural intention to use LMS for the experienced and inexperienced teacher groups (i.e., second research question). Two separate regression analyses were conducted for each teacher group. Satisfaction with Qridi LMS use was the dependent variable for the first regression and behavioural intention to Use Qridi in the future was the dependent variable for the second regression.

## 6 RESULTS

*RQ1*. Are there differences between the experienced and inexperienced teacher groups in terms of their LMS acceptance?

**FIGURE 1:**
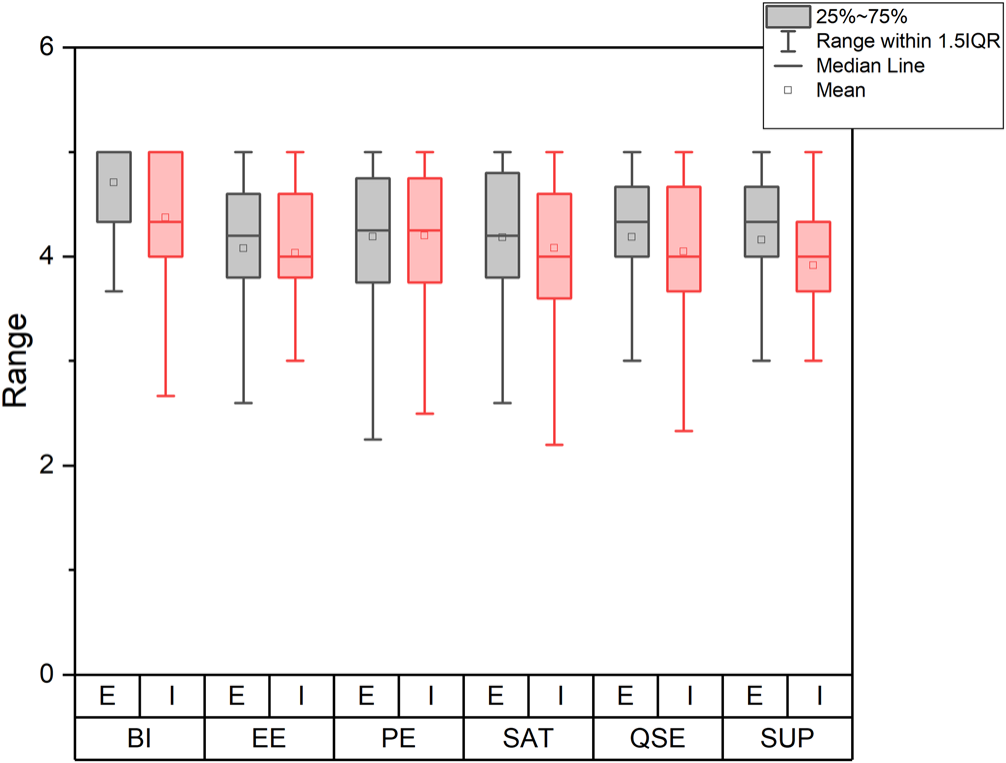
Group scores across the UTAUT dimensions. BI, behavioural intention; E, experienced group; EE, effort expectancy; I, inexperienced group; PE, performance expectancy; QSE, Qridi self‐efficacy; SAT, satisfaction; SUP, support [Colour figure can be viewed at wileyonlinelibrary.com]

According to the independent sample *t* test results, no difference was observed between experienced (Exp) and inexperienced (inExp) teachers in terms of Effort Expectancy (*M*_Exp_ = 4.08; *SD*_Exp_ = 0.60; *M*_inExp_ = 4.03; *SD*_inExp_ = 0.66), Performance Expectancy (*M*_Exp_ = 4.19; *SD*_Exp_ = 0.71; *M*_inExp_ = 4.20; *SD*_inExp_ = 0.62), Qridi self‐efficacy (*M*_Exp_ = 4.19; *SD*_Exp_ = 0.66; *M*_inExp_ = 4.05; *SD*_inExp_ = 0.73) or satisfaction (*M*_Exp_ = 4.18; *SD*_Exp_ = 0.64; *M*_inExp_ = 4.08; *SD*_inExp_ = 0.68). However, the independent samples *t* test showed that experienced teachers (*M*_Exp_ = 4.16; *SD*_Exp_ = 0.76) had higher perceived support compared to the inexperienced teachers (*M*_inExp_ = 3.91; *SD*_inExp_ = 0.67). Further, experienced teachers reported higher Behavioural Intention to use Qridi compared with the inexperienced teachers (*M*_Exp_ = 4.70; *SD*_Exp_ = 0.48; *M*_inExp_ = 4.37; *SD*_inExp_ = 0.69). *t* test results are presented in Table 2, and the group averages are presented in Figure 1.

**TABLE 2:** Independent samples *t* test results for effort expectancy, performance expectancy, Qridi LMS self‐efficacy, satisfaction and behavioural intention

	*t*	*df*	*p*	Mean difference	Partial eta‐squared
Effort expectancy	0.463	194	0.644	0.04311	0.001
Performance expectancy	−0.107	194	0.915	−0.01096	0
Qridi self‐efficacy	1.347	194	0.180	0.13804	0.009
Support	2252	194	0.025	0.24444	0.025
Satisfaction	1.040	194	0.300	0.10217	0.006
Behavioural intention	3.580	105.568	0.001	0.33735	0.075

**FIGURE 2:**
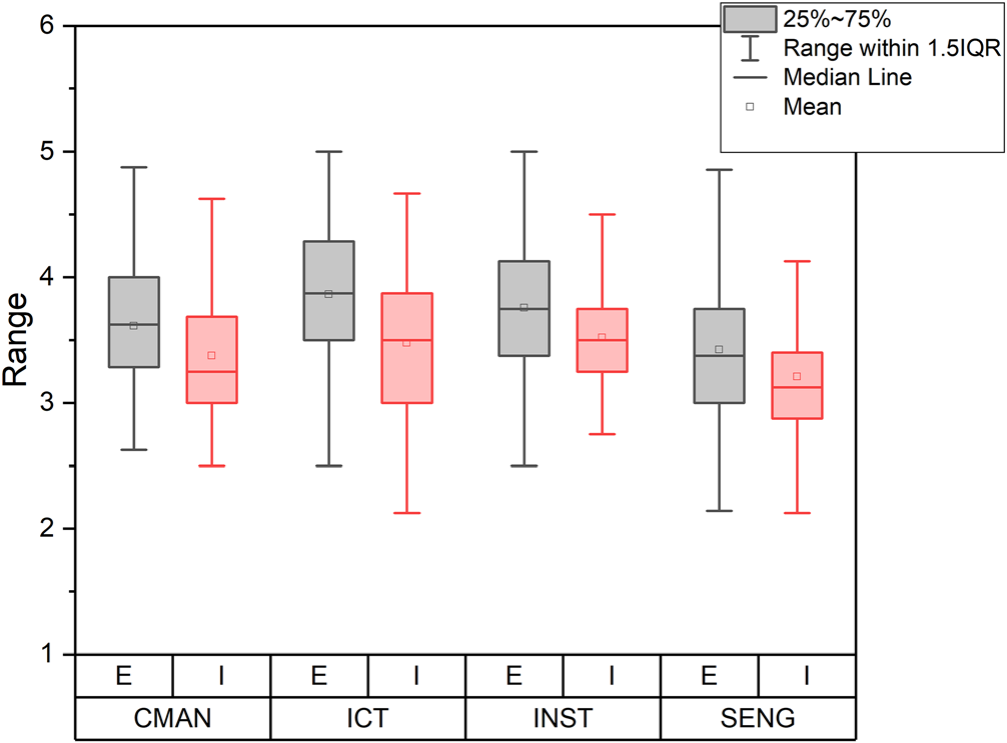
Group scores across online teaching self‐efficacy dimensions. CMAN, classroom management self‐efficacy; E, experienced group; I, inexperienced group; ICT, information and communication technology; INST, instructional strategies self‐efficacy; SENG, student engagement self‐efficacy [Colour figure can be viewed at wileyonlinelibrary.com]

Independent samples *t* tests results further showed that experienced group had higher online teaching self‐efficacy in Student engagement (*M*_Exp_ = 3.42; *SD*_Exp_ = 0.50; *M*_inExp_ = 3.20; *SD*_inExp_ = 0.46), Classroom management (*M*_Exp_ = 3.62; *SD*_Exp_ = 3.37; *M*_inExp_ = 3.38; *SD*_inExp_ = 0.45), Instructional Strategies (*M*_Exp_ = 3.76; *SD*_Exp_ = 0.53; *M*_inExp_ = 3.52; *SD*_inExp_ = 0.46) and ICT skills (*M*_Exp_ = 3.86; *SD*_Exp_ = 0.58; *M*_inExp_ = 3.47; *SD*_inExp_ = 0.59). Table 3 displays the *t* test results. Figure 2 displays group means.

**TABLE 3:** Independent samples *t* test results for the online teaching self‐efficacy dimensions

	*t*	*df*	*p*	Mean difference	Partial eta‐squared
Student engagement self‐efficacy	2.934	193	0.004	0.21488	0.043
Classroom management self‐efficacy	3.399	193	0.001	0.23885	0.056
Instructional strategies self‐efficacy	3.112	193	0.002	0.23761	0.048
ICT self‐efficacy	4.399	191	<0.001	0.38984	0.092

*RQ2*. What are the predictors of LMS acceptance for the experienced and the inexperienced teacher groups?

In the current study technology acceptance was measured with two separate dependent variables. The affective acceptance to the LMS was measured with satisfaction and the behavioural attachment was measured with behavioural intention. Prior to the regression analyses, Pearson's correlation analysis was conducted to see the bivariate relationships among the variables. Results are presented in Table 4.

**TABLE 4:** Correlations between the study variables for the experienced and inexperienced groups

	BI	SAT	EE	PE	QSE	SUP	SENG	CMAN	INST	ICT
BI		0.599**	0.431**	0.466**	0.379**	0.318**	0.208	0.210	0.453**	0.283*
SAT	0.607**		0.720**	0.585**	0.553**	0.254*	0.371**	0.203	0.383**	0.224
EE	0.361**	0.703**		0.515**	0.573**	0.231	0.365**	0.291*	0.436**	0.144
PE	0.604**	0.772**	0.564**		0.567**	0.153	0.293*	0.209	0.452**	0.391**
QSE	0.318**	0.617**	0.645**	0.532**		0.066	0.393**	0.273*	0.393**	0.386**
SUP	0.131	0.113	0.124	0.038	0.115		0.297*	0.233	0.159	0.171
SENG	0.312**	0.397**	0.377**	0.257**	0.177*	0.206*		0.697**	0.649**	0.417**
CMAN	0.278**	0.313**	0.301**	0.168	0.162	0.257**	0.776**		0.701**	0.572**
INST	0.156	0.213*	0.336**	0.081	0.226*	0.231**	0.683**	0.712**		0.659**
ICT	0.153	0.143	0.303**	0.040	0.232**	0.239**	0.447**	0.562**	0.626**	

*Note*: Upper part of the diagonal displays results for the inexperienced group. Lower part of the diagonal displays results for the experienced group.

Abbreviations: BI, behavioural intention; CMAN, classroom management self‐efficacy; EE, effort expectancy; ICT, ICT self‐efficacy; INST, instructional strategies self‐efficacy; PE, performance expectancy; QSE, Qridi LMS self‐efficacy; SAT, satisfaction; SENG, student engagement self‐efficacy; SUP, support.

** Correlation is significant at the 0.01 level (2‐tailed);

* Correlation is significant at the 0.05 level (2‐tailed).

For both teacher groups, a stepwise regression was run with performance expectancy, effort expectancy, Qridi self‐efficacy, support, student engagement self‐efficacy, instructional strategy self‐efficacy, classroom management self‐efficacy and ICT self‐efficacy as the independent variables, and the satisfaction as the dependent variable. Results are presented in Table 5. For the experienced teachers, the significant predictors of satisfaction were performance expectancy (Δ*R* ^2^ = 59.2%), effort expectancy (Δ*R* ^2^ = 10.3%), Student engagement self‐efficacy (Δ*R* ^2^ = 1.2%) and Qridi self‐efficacy (Δ*R* ^2^ = 1%). For the inexperienced teachers, the significant predictors of satisfaction were Effort expectancy (Δ*R* ^2^ = 51.1%) and Performance expectancy (Δ*R* ^2^ = 5.7).

**TABLE 5:** Regression results for satisfaction with Qridi LMS

	Experienced group				Inexperienced group			
	Variable	*B*	*SE B*	*β*	Variable	*B*	*SE B*	*β*
Step 1	(Constant)	1.271	0.219		(Constant)	1.078	0.363	
	Performance expectancy	0.695	0.051	0.772***	Effort expectancy	0.744	0.089	0.720***
	Adjusted *R* ^2^	59.2			Adjusted *R* ^2^	51.1		
	*F*	182.71***			*F*	70.092***		
Step 2	(Constant)	0.400	0.231		(Constant)	0.359	0.414	
	Performance expectancy	0.496	0.054	0.551***	Effort expectancy	0.589	0.098	0.570***
	Effort expectancy	0.418	0.064	0.392***	Performance expectancy	0.320	0.104	0.291**
	Adjusted *R* ^2^	69.5			Adjusted *R* ^2^	56.8		
	*F*	143.736***			*F*	44.404***		
Step 3	(Constant)	0.064	0.266					
	Performance expectancy	0.489	0.053	0.543***				
	Effort expectancy	0.373	0.065	0.349***				
	Student engagement	0.161	0.067	0.126*				
	Adjusted *R* ^2^	70.700						
	*F*	101.451***						
Step 4	(Constant)	−0.112	0.273					
	Performance expectancy	0.455	0.054	0.505***				
	Effort expectancy	0.287	0.074	0.268***				
	Student engagement	0.179	0.067	0.139**				
	Qridi self‐efficacy	0.146	0.063	0.150*				
	Adjusted *R* ^2^	71.700						
	*F*	80.106***						

Abbreviations: *B*, unstandardized regression coefficient; *β*, standardized regression coefficient.

* *p* < 0.05;

** *p* < 0.01;

*** *p* < 0.0001.

A stepwise regression was run to explore the predictors of behavioural intention to use Qridi LMS in the future. Performance expectancy, effort expectancy, Qridi self‐efficacy, support, student engagement self‐efficacy, instructional strategy self‐efficacy, classroom management self‐efficacy and ICT self‐efficacy were included as independent variables in the analysis. Results are displayed in Table 6. For the experienced teachers, the only significant predictors of behavioural intention were performance expectancy (Δ*R* ^2^ = 36%) and Classroom management self‐efficacy (Δ*R* ^2^ = 2.6%). For the inexperienced teachers, the significant predictors of behavioural intention were performance expectancy (Δ*R* ^2^ = 20.5%), Instructional strategies self‐efficacy (Δ*R* ^2^ = 16.4%) and support (Δ*R* ^2^ = 4.1%).

**TABLE 6:** Regression results for behavioural intention to use Qridi LMS

	Experienced group				Inexperienced group			
	Variable	*B*	*SE B*	*β*	Variable	*B*	*SE B*	*β*
Step 1	(Constant)	2.986	0.209		(Constant)	2.176	0.522	
	Performance expectancy	0.411	0.049	0.604***	Performance expectancy	0.523	0.123	0.466***
	Adjusted *R* ^2^	36.000			Adjusted *R* ^2^	20.5		
	*F*	70.186***			*F*	18.067***		
Step 2	(Constant)	2.403	0.307		(Constant)	1.208	0.625	
	Performance expectancy	0.391	0.049	0.574***	Performance expectancy	0.368	0.132	0.328
	Classroom management	0.185	0.073	0.182*	Instructional strategies	0.459	0.178	0.305
	Adjusted *R* ^2^	38.600			Adjusted *R* ^2^	26.9		
	*F*	39.878***			*F*	13.160***		
Step 3					(Constant)	0.526	0.683	
					Performance expectancy	0.342	0.29	0.305*
					Instructional strategies	0.420	0.174	0.279*
					Support	0.237	0.108	0.227*
					Adjusted *R* ^2^	31.000		
					*F*	10.884***		

Abbreviations: *B*, unstandardized regression coefficient; *β*, standardized regression coefficient.

* *p* < 0.05;

*** *p* < 0.0001.

## 7 DISCUSSION

The aim of this study was to investigate and compare technology acceptance of two groups of Finnish K‐12 teachers (i.e., experienced and inexperienced Qridi LMS users) in the context of online teaching during Covid‐19 pandemic. The experienced group had prior experience in teaching with a specific LMS before the pandemic. The inexperienced group had no prior experience with the LMS, but they started using Qridi during the pandemic. Both groups used the LMS for online teaching during the pandemic. Based on the UTAUT framework (Venkatesh et al., 2003), a questionnaire (Chao, 2019) was administered to participants to explore their perceptions about the LMS use in terms of performance expectancy, effort expectancy, Qridi LMS self‐efficacy, support, satisfaction and behavioural intention. In addition, participants' general self‐efficacy beliefs about their online teaching were measured in terms of their beliefs how they can provide support for student engagement, conduct classroom management and use instructional strategies and use ICT in teaching.

No difference was evidenced between the experienced and inexperienced teachers in terms of their performance expectancy, effort expectancy, Qridi LMS self‐efficacy and satisfaction. These findings indicate that inexperienced teachers' perceptions about the LMS's performance benefits for teaching activities, its ease of use and their self‐efficacy in using it has reached to the level of experienced teachers in a rather short time period. In addition, inexperienced teachers were equally satisfied with the LMS as experienced teachers. These findings can be explained from several aspects. From an information systems design perspective, studies have shown that the quality of system design (i.e., technical infrastructure and user experience) have a positive effect for performance expectancy, effort expectancy and satisfaction in LMS use (Alsabawy et al., 2013; Kintu et al., 2017; Mohammadi, 2015). Based on this, it can be argued that the system design of LMS was not a barrier for inexperienced teachers during their teaching in times of Covid‐19 pandemic. From a contextual/historical perspective, the Covid‐19 pandemic have triggered extraordinary circumstances in education landscape. The remote/online teaching during the pandemic did not leave much option for teachers other than using LMSs or other digital platforms for teaching. According to the common view, top‐down or forced technology adoption processes might face resistance by the adopters (Rogers, 2003). Therefore, many of the schools are embracing bottom‐up technology adoption processes by including teachers, and in some cases also students, in planning and decision‐making processes of the technological affordances in use (Hauge & Norenes, 2015). Even though bottom‐up approach is involving teachers to the development work, it seems that in general it has not largely affected on teachers' proactive development. For example, Petko et al. (2015) found no difference between teachers' participation in professional development activities among the Swiss schools that employed top‐down or bottom‐up technology adoption. Further, no difference was observed between the schools with top‐down and bottom‐up technology adoption in terms of ICT use in the classrooms (Petko et al., 2015). Complimenting such findings, the current study showed that contextual/historical circumstances might impact teachers' technology acceptance in forced adoption conditions or in emergency remote teaching. That is, teachers might be willing to accept a specific teaching technology more easily in times of unexpected events (Barbour et al., 2020). This conclusion is supported by Hong et al. (2020)'s study that reported a positive relationship between high value to students and acceptance of ICT policy among the Taiwanese teachers.

Inexperienced teacher group reported receiving less support for their online teaching than the experienced group did. A plethora of studies have underlined the importance of support in effective integration of technology in schools (Ertmer et al., 2012; Liu et al., 2017). Support can be in the form of technical assistance about using specific ICT tools or in terms of pedagogical guidance about how to implement ICT to facilitate meaningful learning experiences (Vanderlinde & van Braak, 2010). Considering that teaching in K‐12 settings is traditionally organized in a face‐to‐face format, the literature mostly discusses about means to support ICT integration in actual classrooms. However, Covid‐19 pandemic have moved the K‐12 education to a new territory. It can be assumed that fully online teaching was a new phenomenon for both teacher groups examined in the current study as well as for their students. However, our findings showed that the experienced teacher group in the current study might have benefited from the pre‐existing support structures in their schools. The pre‐existing support structures in the schools of experienced teachers could be the school administration who has been familiar with the LMS, other teachers who have varying knowledge and experience with the LMS or face‐to‐face training activities that took place before the pandemic. The experienced group might have received higher support for their online teaching during the pandemic from such resources when they faced technical or pedagogical challenges in teaching with the LMS. However, the inexperienced teachers did not have much of these opportunities. For example, school administration and other teachers in their schools were not familiar with the LMS either before the pandemic hit. No face‐to‐face training opportunities were provided to the inexperienced teachers. Thus, it can be assumed that dealing with the technical and pedagogical issues about the LMS was not as straightforward for the inexperienced teachers compared to the experienced teachers.

Overall, the current study is in line with the aforementioned previous research that highlights the importance of building sustainable support to facilitate effective ICT integration in schools (Barbour et al., 2020; Beaunoyer & Dup, 2020; Klapproth et al., 2020). Prior research has evidenced that one of the major challenges for the teachers during the COVID19 remote teaching was the lack of sufficient technical and social support (Klapproth et al., 2020). Since the lack of support potentially increases feelings of stress (e.g., Kyriacou, 2001; Punch & Tuetteman, 1996), it is fundamentally important to explore in more details of the possible characteristics of sustainable support in remote teaching. This would provide information what are the support mechanisms that facilitate teachers to cope with the challenging remote teaching times and from where and how teachers seek support.

The teachers who have been using the LMS before the pandemic had displayed higher levels of online teaching self‐efficacy across student engagement, classroom management strategies, instructional strategies and ICT dimensions. It has been argued that teachers' digital competency development occurs gradually based on their use of ICT for everyday teaching purposes (Blau & Shamir‐Inbal, 2017; Vanderlinde & van Braak, 2010). Supporting this view, several empirical studies have found that teachers' daily experiences with the technology develops their confidence in technology use in their classrooms (Liu et al., 2017; Miranda & Russell, 2012). In accordance with the previous research, the current study shows that providing opportunities for teachers to use ICT in their teaching can improve their online teaching self‐efficacy in the long term. Further, it can be argued that Covid‐19 pandemic might have a positive impact on improving self‐efficacy of teachers in using ICT for pedagogical purposes. The pandemic has pushed teachers to embrace LMSs or other online teaching platforms regardless of whether they would like to use them or not. The pandemic does not seem to be eradicated in the short‐term. This means that teachers across the world will keep using teaching online technologies for some time. Thus, it is possible that the continuous online teaching during the pandemic might improve teachers' self‐efficacy beliefs about online teaching and facilitate a faster uptake of technology enhanced learning in K‐12 schools. Future research should test this assumption.

For both teacher groups, experienced and inexperienced, performance expectancy and effort expectancy together explained the most variance on satisfaction with the LMS. However, performance expectancy was the strongest predictor of satisfaction for the experienced group, whereas it was effort expectancy for the inexperienced group. In general, positive beliefs regarding the effectiveness of a learning technology (i.e., perceived usefulness or performance expectancy) has been found to facilitate higher satisfaction with technology enhanced learning environments than effort expectancy (i.e., ease of use) (Al‐Samarraie et al., 2018; Hung et al., 2011; Jin, 2014; Joo et al., 2018; Tawafak et al., 2018). Several studies have also shown that ease of use could only have an indirect effect on satisfaction through the mediation of perceived usefulness (Barrio‐García et al., 2015; Joo et al., 2016; Liao et al., 2009; Pozón‐López et al., 2020). It should be noted that past studies have mostly dealt with a single user profile. Extending previous work, the current study showed that the impact of performance expectancy and effort expectancy on satisfaction might be different on different teacher groups. That is, inexperienced teachers base their LMS satisfaction mostly on the effort that is necessary to use the system, whereas experienced teachers base their satisfaction beliefs on the performance advantage offered by the system. The current findings have implications for LMS design. In order to attract inexperienced users, LMSs should focus on building simple systems that enable users to complete essential tasks with little effort. For this purpose, some complex features of the system can be, for example, hidden from the inexperienced users. Users could be allowed to activate complex features later on as they improve their skills in using the LMS. To our knowledge, no research has been conducted on designing customizable LMSs that allows for gradual increment of system complexity and performance. Future research should explore this opportunity. Our findings further showed that student engagement self‐efficacy and LMS self‐efficacy together explained around 2% variance on satisfaction in the experienced group. Considering the low variance, their practical impact on satisfaction is arguable. Thus, we are cautious in claiming a relationship between self‐efficacy and satisfaction.

Several studies have shown that both performance expectancy and effort expectancy have direct influence on teachers' technology acceptance (Chen & Tseng, 2012; Mazman Akar, 2019; Scherer & Teo, 2019). However, in some studies effort expectancy had an indirect effect on behavioural intention either through the mediation of perceived usefulness (Hu et al., 2003; Teo et al., 2019) or attitude (Scherer et al., 2019). There are also studies that reported no direct or indirect relationship between effort expectancy and behavioural intention (Šumak & Šorgo, 2016). In the current study, performance expectancy was the strongest predictor of behavioural intention for both the experienced and inexperienced teachers. However, no direct effect of effort expectancy was found on behavioural intention. Earlier studies have argued that individuals would not accept a technology simply because it requires little effort to use it (Hu et al., 2003; Keil et al., 1995). Supporting this, the current findings imply that both experienced and inexperienced teachers are willing to use the LMS if they believe that it offers significant advantage in terms of their teaching practice. Therefore, technology integration efforts in education should involve developing positive beliefs among the teachers about the usefulness of instructional technologies. This can be achieved by, for example, providing demonstrations, models and examples to teachers about how to use a specific technology most effectively in teaching.

Online teaching self‐efficacy was another significant predictor of behavioural intention to use LMS although the dimensions that predicted behavioural intention was different for the experienced (i.e., classroom management) and inexperienced teachers (i.e., instructional strategies). The current findings are in accordance with the previous work that underlined the importance of teachers' digital competencies in taking advantage of technology enhanced learning (Kirschner & Selinger, 2003; Straub, 2009). Teachers' technology acceptance does not solely rely on the affordances of a specific technology. Rather, it is a complex process that also includes teachers' self‐efficacy beliefs, and motivational attributes (Scherer & Teo, 2019). In the current study, the targeted self‐efficacy, that is the efficacy in using a specific technology (i.e., Qridi LMS), did not predict behavioural intention in any of the teacher groups. Therefore, it can be argued that technology integration initiatives in educational contexts should go beyond developing competencies of teachers in using specific technologies. Rather, the initiatives should include more holistic approaches and develop teachers' digital competencies in seamless integration of learning technologies aligned with effective pedagogical approaches (Häkkinen et al., 2017; Valtonen et al., 2021).

Finally, support was a significant predictor of behavioural intention only for the inexperienced teachers. The significance of support on behavioural intention disappeared among the teachers who have experience in using the LMS for some time. Previous studies highlight that providing support to teachers is crucial for successful technology integration (Ertmer, 1999; Liu et al., 2017; Vanderlinde & van Braak, 2010). Contributing to such findings, the current study showed that providing support to teachers specifically at the early stages of technology adoption is important for successful technology integration in education. This support can be in the form of offering training activities to teachers, providing them resources (e.g., software, tool and guidelines) or providing them technical support. In time of the pandemic, providing technological support to teachers is not only important for effectiveness of online remote teaching. Studies have reported significant associations between teaching efficacy and well‐being of teachers (Molero Jurado et al., 2019; Putwain & von der Embse, 2019). With the start of the pandemic, many K‐12 teachers have found themselves in the online teaching territory they are not much familiar with. The challenges they face during adaptation to online remote teaching imposes high stress and emotional burden on teachers (Marek et al., 2020). It has been found that lack of online teaching skills is one of the main contributors of stress among the teachers (Khlaif et al., 2020). Therefore, providing effective technology integration support to teachers can be considered as crucial for also supporting their well‐being. This can be viewed also as a collaborative act, where teachers in a stressful working situation would gain from the peer/collegial support.

## 8 LIMITATIONS

The current study explores the direct relationships between the variables of interest. Due to sample size, it was not possible to investigate the indirect relationships among the variables across the groups. Future research should address this limitation by collecting data from a bigger sample of teachers and testing structural equation models for both groups. This is a cross‐sectional study. Thus, the current findings inform about two groups of teachers' technology acceptance beliefs at a specific time. A longitudinal approach that measures teachers' beliefs multiple times over time would have been a better approach. This would provide a fine‐grained look in how teachers' beliefs change as they experience specific educational technologies. The current study is based on a modest sample size. Although the sample size allows us to run relevant parametric tests to answer our research questions, the current findings might not be generalized to teacher population in Finland or elsewhere. Further, majority of the participants were female in the current study, which characterize gender distribution within the teaching profession in Finland where the great majority of primary school teachers are females. Due to small male sample, we could not include gender as a distinct variable in the statistical analyses. However, gender has been reported as a significant construct in teachers' technology adoption process (Teo, 2014). Therefore, future studies should address this limitation by recruiting higher samples from both males and females. Another limitation of this study is that it employed convenience sampling of Qridi LMS users. Eventually, majority of the participants who responded to the survey appeared to be the teachers who had previous experience in Qridi LMS before the pandemic. It would have been better if the sample size of the inexperience teachers were proportionate to the experience teachers. In the current study, the frequency of Qridi LMS usage was not measured. Exploring behavioural usage patterns of LMS could provide valuable insights about understanding the technology acceptance among teachers with and without prior LMS experience when the pandemic hit the education landscape.

## 9 CONCLUSION

The ongoing Covid‐19 pandemic has caused an unprecedented shift towards online remote teaching. Hence, it is important to understand the impact of such abrupt shift on teachers' online teaching technology acceptance. Drawing on this, the current study explored the LMS acceptance of two groups of Finnish K‐12 based on their prior experiences with a specific LMS. The first group had been using the LMS before the pandemic (i.e., experienced group). The second group started using it during the pandemic (i.e., inexperienced group). The current study raises several key conclusions. First, inexperienced teachers displayed similar levels of technology acceptance with the experienced teachers although they had to adopt the LMS in a forced manner due to the pandemic. These findings imply that if teachers are convinced about the necessity of using a specific technology in a specific condition (e.g., pandemic) they will accept it regardless of whether it is imposed on them or not. Thus, we suggest school administrators and policy makers to put effort in developing open communication with their teachers about the importance of using technology in their practice rather than solely focusing on developing facilitating conditions for technology use (i.e., the technological infrastructure and training). From a practical perspective, the existing study addresses an important implication about LMS design. We have found that ease of use is a significant factor for inexperienced teachers' satisfaction with the LMS. This calls for future research on user experience design in LMSs that specifically focuses on facilitating easy‐to‐use interfaces for inexperienced teachers. Another key contribution of this study is that technology integration in education is a developmental process that is impacted by multiple factors. These factors might be related to the technology in use, teachers' general or targeted digital competencies in teaching with technology, and organizational support provided to teachers. Further, teachers' prior experiences with the technology can be interrelated with these factors. Thus, we suggest that future research should study teachers' technology acceptance by looking at how technology acceptance constructs vary among teachers with different prior experience rather than studying them as a unified profile.

## CONFLICT OF INTEREST

The authors declare no conflicts of interest.

## Data Availability

The data that support the findings of this study are available from the corresponding author upon reasonable request.
